# The influence of iron on the proteomic profile of *Chromobacterium violaceum*

**DOI:** 10.1186/s12866-014-0267-6

**Published:** 2014-10-20

**Authors:** Daniel C Lima, Fábio T Duarte, Viviane KS Medeiros, Diogo B Lima, Paulo C Carvalho, Diego Bonatto, Silvia R Batistuzzo de Medeiros

**Affiliations:** 1 Laboratório de Biologia Molecular e Genômica, Centro de Biociências, Universidade Federal do Rio Grande do Norte (UFRN), Campus Universitário s/n, Lagoa Nova, Natal, RN 59072-970 Brazil; 2 Instituto Federal de Educação, Ciência e Tecnologia do Rio Grande do Norte, São Gonçalo do Amarante, RN Brazil; 3 Universidade Federal do Vale do São Francisco, Petrolina, PE Brazil; 4 Laboratório de Proteômica e Engenharia de Proteínas, Instituto Carlos Chagas, Fiocruz, PR Brazil; 5 Departamento de Biologia Molecular e Biotecnologia, Centro de Biotecnologia, Universidade Federal do Rio Grande do Sul (UFRGS), Avenida Bento Gonçalves, 9500–Prédio 43421, Caixa Postal 15005, 91509-900 Porto Alegre, RS Brazil

**Keywords:** Energetic metabolism, Coordinated adaptation, Sod

## Abstract

**Background:**

*Chromobacterium violaceum* is a bacterium commonly found in tropical and subtropical regions and is associated with important pharmacological and industrial attributes such as producing substances with therapeutic properties and synthesizing biodegradable polymers. Its genome was sequenced, however, approximately 40% of its genes still remain with unknown functions. Although *C. violaceum* is known by its versatile capacity of living in a wide range of environments, little is known on how it achieves such success. Here, we investigated the proteomic profile of *C. violaceum* cultivated in the absence and presence of high iron concentration, describing some proteins of unknown function that might play an important role in iron homeostasis, amongst others.

**Results:**

Briefly, *C. violaceum* was cultivated in the absence and in the presence of 9 mM of iron during four hours. Total proteins were identified by LC-MS and through the PatternLab pipeline. Our proteomic analysis indicates major changes in the energetic metabolism, and alterations in the synthesis of key transport and stress proteins. In addition, it may suggest the presence of a yet unidentified operon that could be related to oxidative stress, together with a set of other proteins with unknown function. The protein-protein interaction network also pinpointed the importance of energetic metabolism proteins to the acclimatation of *C. violaceum* in high concentration of iron.

**Conclusions:**

This is the first proteomic analysis of the opportunistic pathogen *C. violaceum* in the presence of high iron concentration. Our data allowed us to identify a yet undescribed operon that might have a role in oxidative stress defense. Our work provides new data that will contribute to understand how this bacterium achieve its capacity of surviving in harsh conditions as well as to open a way to explore the yet little availed biotechnological characteristics of this bacterium with the further exploring of the proteins of unknown function that we showed to be up-regulated in high iron concentration.

**Electronic supplementary material:**

The online version of this article (doi:10.1186/s12866-014-0267-6) contains supplementary material, which is available to authorized users.

## Background

*Chromobacterium violaceum* is a mobile bacillus, a facultatively aerobic and free-living organism that is associated with opportunistic infections in immunosuppressed individuals. Its ability to withstand various antibiotics poses this organism as responsible to a considerable number of deaths [1,2]. Research on pharmaceutical applications of *C. violaceum* have been carried out since the 1970’s with reported production of antitumor peptides, compounds with analgesic and antibiotic capability [3-5], and synthesis of biodegradable polymers [6]. *C. violaceum’s* hallmark is the production of the purple pigment violacein that has innumerous therapeutic properties such as anti-tumoral activity [7].

Pharmacological and industrial attributes motivated the scientific community to sequence its approximately 4.7 Mbp genome [8]. To date, approximately 40% of its ORFs remain with unknown functions which make this organism a target for prospecting genes with biotechnological properties. Previous reports have described several key aspects of *C. violaceum*, such as having great metabolic versatility presenting several ORFs related to osmotic stress, response to heat, oxidative stress, DNA repair, and a large number of proteins involved in iron metabolism [9,10].

In general, microorganisms need iron to survive; it is essential in key biological processes such as cellular respiration, electron transfer, gene regulation, DNA synthesis [11,12] and is known to play key roles in metabolism of various pathogenic microorganisms [13-16]. Despite its importance in catalysis of biological processes, an excess of iron can damage cells through the Fenton reaction in which ferrous ions react with hydrogen peroxide and produce hydroxyl radical [17]. Thus, high concentrations of this metal can lead to oxidative stress which causes damage to biomolecules and ultimately the death of the organism or cell [17].

The aim of this study was to compare the proteomic profiles of *C. violaceum* cultivated in absence and presence of iron. Taken together, a further comprehension on the metabolism of this metal in pathogenic organisms can aid in the discovery of therapeutic targets and further understanding in the biology of its adaptation mechanisms. Briefly, our results showed an increased synthesis of proteins mainly related with the tricarboxylic acid cycle (TCA), transportation, oxidative stress, and lists key proteins possibly linked to its adaptation in an iron rich medium. In addition, under the light of proteomic and RT-PCR data, we identified a new non-characterized *operon* possibly related to oxidative stress, composed with *sodB2* and two other genes of unknown function.

## Methods

### Culture conditions and treatment with FeSO_4_

Isolated colonies of *Chromobacterium violaceum*, ATCC 12472 strain, were inoculated in liquid Luria Bertani (LB) medium for 16–18 hours at 28°C and shaken at 200 rpm.

The treatment was performed in an Erlenmeyer flask diluting the pre-cultivated bacteria with liquid LB medium (1:10) in a final volume of 100 mL. The FeSO_4_ solution was prepared at an initial concentration of 500 mM and was further filtered with 0.22 μm GHP membrane disc filters [Acrodisc] and added at a final concentration of 9 mM. This concentration was chosen after a screening of treatment concentrations in which at only 9 mM of iron we observed different patterns of proteins synthesis at 1D-SDS-PAGE, in contrast to the negative control as well as previous results of our group that have demonstrated the high resistance of *C. violaceum* to iron (data not shown). The negative control consisted of *C. violaceum* grown in a LB broth. The treatment exposed the culture for four hours under the same conditions as described above. A measurement of *C. violaceum* growth was performed between 0–4 hours and the optical density was read at 600 nm in a 96-well spectrophotometer. The negative control and the experimental conditions were performed in biological triplicate.

### Total intracellular iron measurement

Total intracellular iron concentration was estimated as described at Barbehenn et al. [18]. First, *C. violaceum* cultures (100 mL) were grown until reaching 0.4–0.5 OD when the treatment began. After the four-hour treatment, the samples were centrifuged at 4°C, 2700 g for 15 min. Then, samples were washed with 50 mM EDTA, followed by another wash with ultrapure water. Finally, the phenanthroline assay was performed as described by Barbehenn et al. [18]. The negative control and the experimental condition were performed in biological triplicate.

### Protein extraction

After four hours of treatment, the samples were centrifuged at 2880 g and 4°C for 20 minutes. The supernatant of all samples (including negative control) were discarded and then the samples were washed in 50 mM EDTA pH 8.0 and centrifuged under the same conditions previously mentioned. Then the samples were washed once more in 10 mM Tris–HCl, pH 8.5 solution and centrifuged at 2880 g, 4°C for 25 minutes. Cells were lysed in a 300 μL – 400 μL extraction buffer containing 7 M urea, thiourea 1 M, 50 mM DTT, 0.5% CHAPS, and 30 mM Tris pH 8.5. Proteins were precipitated by adding 1 mL of acetone, vortexing the samples and then centrifuging at 10,000 g, 4°C for 3 minutes. This process was performed a second time, and the sample was centrifuged again for 5 minutes. Finally, the proteins were solubilized in the same extraction buffer (300 μL–900 μL).

### Antioxidant activity evaluation of *Chromobacterium violaceum*

In order to evaluate if the iron treatment promotes oxidative stress in *Chromobacterium violaceum*, antioxidant enzymes catalase (CAT) and superoxide dismutase (SOD) activities were measured on total protein extracts. Catalase Assay Kit 707002 and Superoxide Dismutase Assay Kit 706002 (Cayman Chemical, Ann Arbor, MI) were used according to manufacturer’s recommendations to quantify the catalase and superoxide dismutase activity level, respectively. The total antioxidant activity was evaluated using the Antioxidant Assay kit from Sigma-Aldrich (CS0790) according to manufacturer’s instructions. The protein concentration values were used to normalize the enzyme activity. The experiment was performed in biological triplicate.

### SDS-PAGE

The total protein extracts were quantified by the Bradford method [19] and 20 μg of protein from each sample was resolved on a polyacrylamide gel under denaturing conditions (SDS-PAGE) at 12%. The marker used was the Precision Plus Proteins™ WesternC™ Standard from Bio-Rad. The gel was stained using the Coomassie Colloidal from Sigma-Aldrich.

### In gel digestion and peptides extraction

Following electrophoresis, each lane was excised in nine fragments according to the protein’s density. All the proteins from each lane were excised and each group of proteins was analyzed independently by mass spectrometry (see below). Proteins were extracted and digested from these fragments as in accordance to the revised Shevchenko et al. protocol [20]. The dye and the SDS were quickly removed by washing the fragments three times in 50% acetonitrile solution (ACN) and 10 mM ammonium bicarbonate. Then the bands were dehydrated in ACN at 100%, reduced with 10 mM dithiothreitol (DTT) at room temperature and alkylated with 50 mM iodacetamide (IAA) in a dark environment. Then, the bands were washed again with 100 mM ammonium bicarbonate, dehydrated with 100% ACN, and rehydrated with 100 mM ammonium bicarbonate; this was done twice. The bands were then hydrated in a trypsin solution (Trypsin Gold, Mass Spectrometry Grade from Promega [v5280]) prepared according to manufacturer’s instructions. 35–50 μL of trypsin at 20 μg/mL was added to the samples kept on ice. Then we added 50 mM of ammonium bicarbonate, sufficient to cover the bands during incubation at 37°C for 16–18 hours.

For peptide extraction, 10–30 μL of 5% formic acid were added to the bands. After 10 minutes of incubation at room temperature, the supernatant containing peptides was transferred to another tube. Then a second extraction solution (5% formic acid and 50% ACN) was added in enough volume to cover the bands. The supernatant was transferred to a previously prepared tube containing the already extracted peptides. The extraction of the peptides performed with the second solution was repeated once again. Finally, the solution containing the digested peptides was concentrated in Eppendorf’s *Concentrator Plus*.

### Mass spectrometry data acquisition

After the *in gel* digestion, the samples were loaded onto the liquid chromatography NanoAcquity UPLC system (Waters) connected with an ESI-Q-Tof *premier* (Waters) mass spectrometer. The tryptic peptides from each sample (4.5 μL) were separated on a BEH130-C18 column (100 μm × 100 mm) at a 600 nL/min flow rate. The gradient varied from 2 to 98% ACN in 0.1% formic acid for 45 minutes. The instrument’s data acquisition mode was set to a data dependent “top three” and controlled using MassLynx v.4.1.

### Protein identification

The ProLuCID search engine v 1.3 [21] was used to compare experimental spectra against those theoretically generated from *C. violaceum* ATCC 12472 sequence downloaded from Uniprot in January 2013, plus those from 127 common contaminants to proteomic experiments (e.g., Keratin, BSA, etc.). The search was limited to tryptic and semi-tryptic peptide candidates; carbamidomethylation and oxidation of methionine were imposed as fixed and variable modifications, respectively. The search engine accepted peptide candidates within a 50-ppm tolerance from the measured precursor m/z and used the XCorr and Z-Score as the primary and secondary search engine scores, respectively.

The validity of the Peptide Sequence Matches (PSMs) was assessed using the Search Engine Processor (SEPro) v.2.2.0.1 [22]. Briefly, identifications were grouped by charge state (+2 and ≥ +3) and then by tryptic status (tryptic or semi-tryptic), resulting in four distinct subgroups. For each group, the XCorr, ZScore, DeltaCN, and DeltaMass values were used to generate a Bayesian discriminant function. The identifications were sorted in a non-decreasing order according to the discriminator score. A cutoff score was established to accept a false-discovery rate (FDR) of 1% based on the number of decoys. This procedure was independently performed on each data subset, resulting in a false-positive rate that was independent of tryptic status or charge state. Additionally, a minimum sequence length of six amino acid residues was required. Results were post processed to only accept PSMs with less than 10 ppm and proteins supported by two or more independent evidences (e.g., identification of a peptide with different charge states, a modified and a non-modified version of the same peptide, or two different peptides). This last filter led to a 0% FDR in all search results for all our sample analyses.

### Differential proteomics and functional analysis

The PatternLab’s updated ACFold module was employed to pinpoint differentially expressed proteins between the control and iron exposed condition [23,24]. The revised ACFold module presents increased sensitivity under the Benjamini-Hochberg q-value [25] bound by applying a variable fold-change that varies with the AC-test p-value as a power law [24].

Proteins uniquely identified in one condition (control or iron) were pinpointed according to PatternLab’s Approximate Area Proportional Venn Diagram module. To better cope with the limitations from undersampling, we only considered proteins identified in two replicates of each condition, and not found in any replicates of the other condition.

The functional categorization of the proteins was assessed using PatternLab’s Gene Ontology Explorer (GOEx) module [26]. Our data analysis used the gene ontology database (OBO v1.2 - http://www.geneontology.org/GO.format.obo-1_2.shtml), downloaded February 16th, 2013 and the *C. violaceum* gene ontology annotation in the Uniprot text file format of its protein sequences.

### Functional domain analysis

The protein sequences were compared [27] against the protein database from NCBI for automatic annotation and functional domains were predicted using the Conserved Domain Database (CDD) [28].

The structural prediction of hypothetical ORFs was performed using the server for protein homology detection HHpred (data not shown) [29]. FASTA sequences from each hypothetical protein were submitted to the server and the PDB database from February 23rd of 2013 was used. No specific organism proteome was selected, the method for multiple sequence alignment (MSA) generation was HHblits [30], up to 3 MSA generation iterations was used, the secondary structure score was applied, and the alignment mode was set to local.

### Protein-protein interaction network analysis

The metasearch tool STITCH 3.1 (http://stitch.embl.de) was used to estimate protein-protein interaction networks related to iron response. STITCH is a tool used to explore known and predicted interactions between proteins, and chemical or physical agents. These agents interconnected by evidences derived from experiments, databases, and literature. One network was generated composed of proteins that were identified exclusively in the iron treatment or having an increased expression after being exposed to the metal. As the interaction targets were derived from experimental data, a high confidence index (0.700) was used to generate the networks. All prediction methods were activated: neighborhood, gene fusion, co-occurrence, co-expression, experiments, and databases text mining.

The assembled network was exported to be subsequently analyzed in Cytoscape 2.8.2 and Cytoscape 3.0 [31]. The former was used to calculate the centrality indexes and the later for the remaining analysis. The most relevant proteins sub-networks were selected using the Cytoscape MCODE v. 1.4.0 plugin (Molecular Complex Detection) [32]. The MCODE analysis parameters were: loops inclusion; degree cutoff of 2; haircut option enabled (which leads to deletion of nodes cluster with a single connection); fluff option enabled, node score cutoff at 0.2; K-core at 2, and maximum depth of 100. The only clusters used were those in which the MCODE index score was greater or equal to 2.5.

The network centrality calculations were computed from local networks and topologies. The network’s bottlenecks were identified through a Betweenness vs. Node Degree chart with the values generated by the CestiScaPe 1.21 plugin installed at Cytoscape 2.8.2. Betweenness indicates the extent to which a particular node is among all other nodes in a network and usually shows the influence of this node on information propagation within the network [33,34]. Node Degree corresponds to the number of connections that a particular node has with other adjacent nodes. High node degree levels are called “hubs” [35]. Thus, a particular node with high node degree and betweenness values represents a bottleneck, or a protein that interconnects many biological processes [34].

### Total RNA extraction from *C. violaceum* and cDNA synthesis

Isolated colonies of *C. violaceum* were cultured in absence and the presence (9 mM) of iron in the previously mentioned conditions. Further, 2 mL of the culture was used to extract and purify total RNA using the *RNAspin Mini Isolation* kit according to manufacturer’s instructions (GE, catalog number 25-0500-72).

Once extracted, the total RNA was used to synthesize cDNA with random primers with the *High capacity cDNA reverse transcriptase* kit according to manufacturer’s instructions (Applied Biosystems, catalog number 4368814).

### RT-PCR analysis

The Reverse Transcriptase Polymerase Chain Reaction (RT-PCR) was used to validate the expression of the hypothesized operon as a unique transcript. As such, RT-PCR was performed to provide the transcript evidence of regions encompassing the ORFs CV_0869 (the first) and CV_0867 (the last). Primers for the above mentioned genes were designed using Primer 3 (v. 0.4.0) software.

RT-PCR was performed using AmpliTaq Gold® 360 Master Mix (Catalog Number 4398881, Applied Biosystems). One microlitrer of cDNA was used and a final volume of 25 μL was used according to the manufacturer’s instructions. Then, the PCR steps were the following: initial denaturation at 98°C for 5 min, 40 cycles of 98°C for 30 sec, 59.8°C for 30 sec, 72°C for 90 sec and a final extension at 72°C for 7 min were followed. Reactions using water and RNA instead of cDNA were carried out as negative controls. To verify the size of the amplicons, 5 μL of the PCR reaction was loaded on 1% agarose gel and submitted to electrophoresis. The DNA ladder of 1 Kb (Promega) was used as reference. An additional file (Additional file 1) shows the sequence of the primers used in this work.

### Validation of expression by Real-Time quantitative PCR

The quantitative real-time PCR was used to validate the proteome analysis by verifying the expression of the genes CV_0868 and CV_0867, both comprising what we hypothesized as a newly described operon. Seven nanograms of cDNA (produced as mentioned above, from the iron-culture and control) and a final concentration of 0.25 nM of each primer were applied in a 10 μL reaction using Power SYBR® Green PCR Master Mix (Applied Biosystems, 4367659) in an One-Step cycling with the following conditions: 95°C for 3 min, 40 cycles of denaturation at 95°C for 3 sec, annealing/extension at 60°C for 15 sec. The 16S rRNA was used as endogenous control and to normalize the expression of the other two genes. The relative quantification was assessed by ΔCt comparative analysis. The primers were designed using Primer 3 (v. 0.4.0). Primers sequences are described in Additional file 1. Statistical analysis was performed according to the t-test. Results were considered significant for *p* <0.05. Two biological replicates were used.

## Results

### *C. violaceum* growth and intracellular iron estimation

**Figure 1:**
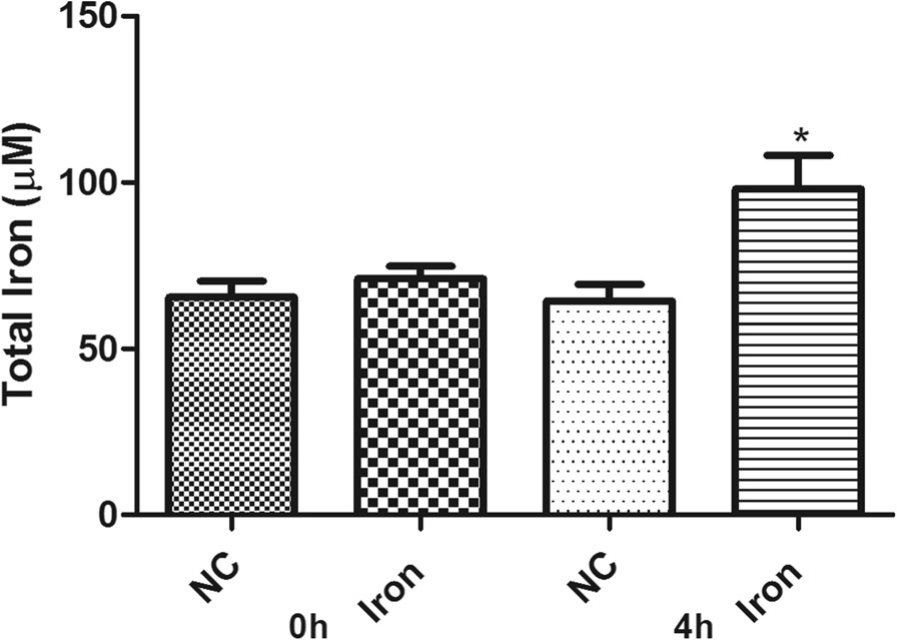
**Intracellular iron concentration of** ***C. violaceum*** **before and after the treatment.** NC = Negative Control.

The phenanthroline assay showed that the treatment is, as expected, leading to a significant increase in the total intracellular iron in *C. violaceum* (Figure 1) when compared with the bacterium grown in the absence of the metal. The OD measurement (Additional file 2) showed that the iron treatment leads to a growth arrest in *C. violaceum* although, from this assay, we cannot see a death tendency caused by the experimental condition, suggesting that this bacterium has a mechanism to withstand this elevated iron exposure.

### *C. violaceum* antioxidant profile

**Figure 2:**
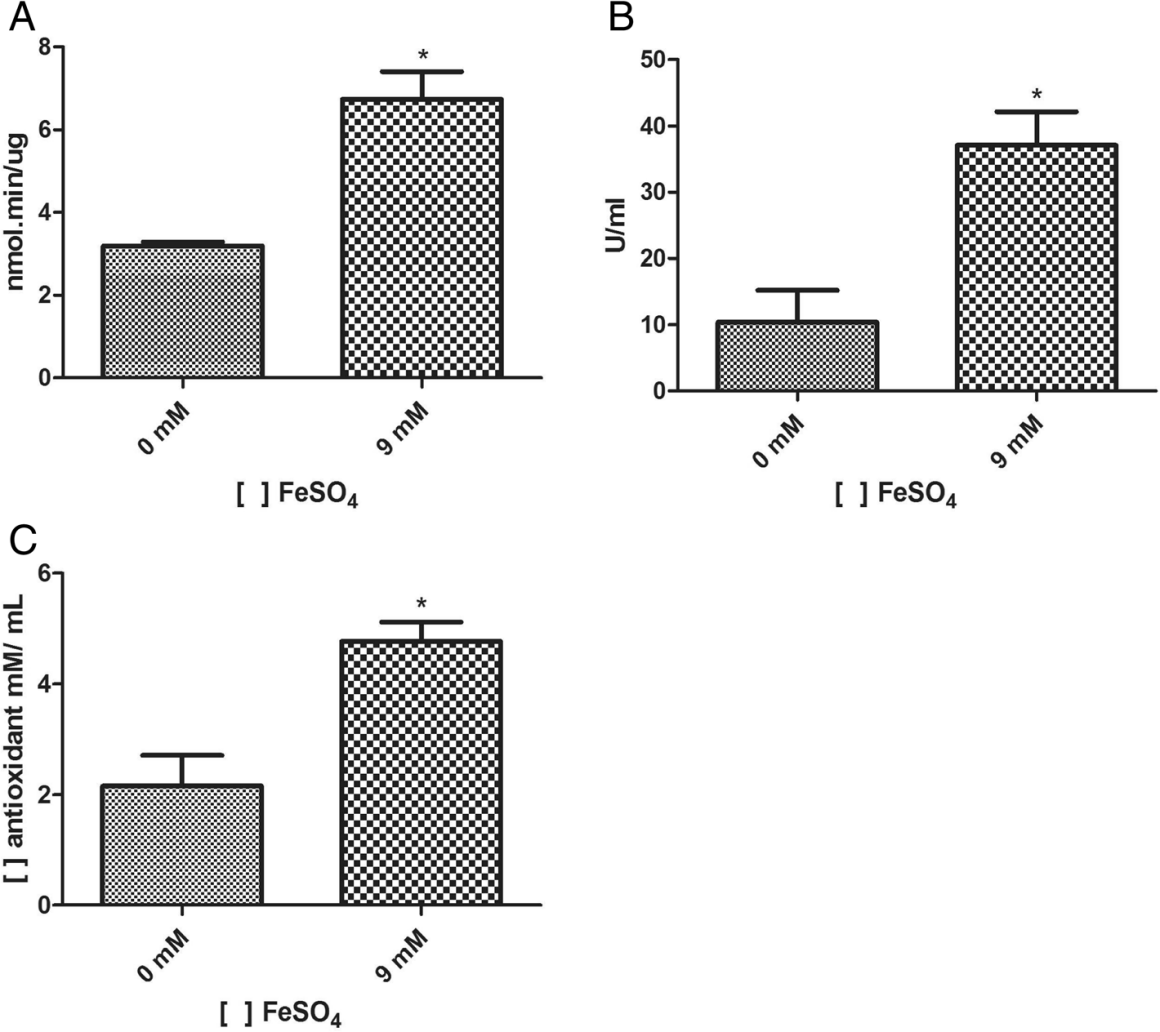
**Antioxidant profile of** ***Chromobacterium violaceum*** **. A)** Catalase activity assay. **B)** Superoxide dismutase activity assay. **C)** Total antioxidant activity assay of the total proteic extract of *Chromobacterium violaceum*.

We evaluated if the iron concentration used in the experiment can induce oxidative stress by assessing the catalase and superoxide dismutase enzymatic activities and the total antioxidant activity from the *C. violaceum* protein extract. The catalase activity (Figure 2A), the superoxide dismutase activity (Figure 2B), and the total antioxidant activity (Figure 2C) increased significantly during the treatment performed with 9 mM iron, which suggests that the presence of metal is inducing an oxidative stress scenario in *C. violaceum*.

### LC-MS/MS analysis

PatternLab’s analysis allowed us to identify 230 proteins found in at least two biological replicates and in both biological conditions of which 28 were only identified in the control and 9 in the iron-responsive proteome. We note that identifying a protein exclusively in a condition does not exclude the hypothesis that it is present in the other condition; it can be below the detection sensitivity or not identified given the random sampling nature and undersampling of our data acquisition strategy. Nevertheless, for the experimental design at hand, identifying proteins on a single condition for two technical replicates suggests a differential expression [36].

The differentially expressed proteins found in the Control versus Iron treatment were accessed according to the updated ACFold module; 45 proteins were pointed as differentially expressed (Blue dots in Additional file 3) (q <0.05). Table 1 lists the differentially expressed proteins while the Additional files 4 and 5 list the proteins identified only in the iron-responsive proteome or control condition, respectively.

**Table 1:** **Differential expressed proteins distributed in functional categories**

**Uniprot identifier**	**Fold change**	**Coverage**	**Description**	**Spectral count**
**Up-regulated**				
**Energetic metabolism**				
Q7NZ60	2.01267122691591	0.3865	Malate dehydrogenase (Mdh)	76
Q7NY63	2.12401648386714	0.4413	Formate C-acetyltransferase (PflB)	139
Q7NZ52	2.52323856021444	0.1701	Citrate synthase (GtlA)	30
Q7NQM5	3.90695002871913	0.2484	Fumarate hydratase class II (AspA)	35
Q7NZ50	3.90695002871913	0.2054	Dihydrolipoamide succinyltransferase E2 component (SucB)	21
**Proteins of unknown function**				
Q7NZQ3	3.32090752441126	0.3605	Putative uncharacterized protein (CV_0868)	26
Q7NQ40	3.71160252728317	0.4051	Putative uncharacterized protein (CV_4300)	20
**Oxidative metabolism**				
Q7NWH0	15.6278001148765	0.2869	Probable aldehyde dehydrogenase	15
Q7NUH0	1.73292138370606	0.3369	Probable alcohol dehydrogenase	61
**Stress response**				
Q7P1C4	10.7441125789776	0.2365	Glutathione S-transferase family protein	10
**Translation**				
Q7NQG5	12.6975875933372	ND	30S ribosomal protein S14	ND
Q7NVZ4	1.65034958109687	0.4815	30S ribosomal protein S2	60
**Transport**				
Q7NZ25	2.4154454569446	0.5273	Probable binding protein component of ABC dipeptide transporter	212
Q7NQ13	3.6278821695249	0.1415	Probable oligopeptide ABC transporter system, substrate-binding protein	24
Q7NQN4	11.7208500861574	0.1116	Outer membrane protein W	11
Q7NSK0	2.05114876507754	0.6229	Porin signal peptide protein	347
Q7NXT7	4.23252919777905	0.2015	Probable amino acid ABC transporter	12
**Others**				
Q7NYA8	1.7992533026996	0.6141	Probable phasin	46
Q7NYB1	2.27905418341949	0.4851	Probable trans-acting regulatory HvrA protein	30
Q7NX40	28.3253877082137	0.2368	Protein kinase	30
**Down-regulated**				
**Energetic metabolism**				
Q7P0K7	−10.238165245516	0.241	Glyceraldehyde-3-phosphate dehydrogenase (GapA)	18
Q7NX09	−3.75399392335588	0.1019	Probable ribonuclease E	13
**Stress response**				
Q7NXP2	−9.21434872096442	0.3333	Thioredoxin	7
Q7NXI3	−2.18117433491428	0.2897	Chaperone protein DnaK	50
Q7NYF6	−13.3096148191708	0.1268	Chaperone protein HtpG	12
Q7NQ87	−13.3096148191708	0.3355	DNA-binding stress protein	15
**Translation**				
Q7NQH5	−9.21434872096442	0.2214	30S ribosomal protein S11	8
Q7NQH1	−9.21434872096442	0.3916	50S ribosomal protein L15	16
Q7NRL5	−4.35122022934431	0.4286	30S ribosomal protein S21	17
Q7NQF6	−2.27514783233689	0.3696	30S ribosomal protein S19	20
Q7NQE5	−2.13295109281584	0.374	50S ribosomal protein L7/L12	27
Q7NQF0	−1.87507970226867	0.4355	Elongation factor G	178
Q7NQH7	−1.82506423941807	0.3639	DNA-directed RNA polymerase subunit alpha	51
Q7NQE6	−1.62879901633209	0.182	DNA-directed RNA polymerase subunit beta	36
Q7NQF3	−1.59971331961188	0.6311	50S ribosomal protein L4	99
Q7NQF4	−13.3096148191708	0.3922	50S ribosomal protein L23	23
P60100	−12.2857982946192	0.2168	50S ribosomal protein L11	16
Q7M7F1	−1.04957291510636	0.6061	Elongation factor Tu	524
Q7NQG9	−2.0476330491032	0.4477	30S ribosomal protein S5	36
Q7NRP0	−10.238165245516	0.1005	Aspartate--tRNA ligase	9
**Proteins of unknown function**				
Q7NQ36	−11.2619817700676	0.3807	Putative uncharacterized protein (CV_4304)	10
**Others**				
Q7NXX7	−9.21434872096442	0.0967	N-succinylglutamate 5-semialdehyde dehydrogenase	8
Q7NXU5	−2.51300419662666	0.2443	Acetate kinase	26

All the proteins listed below has a q value <0.05.

**Figure 3:**
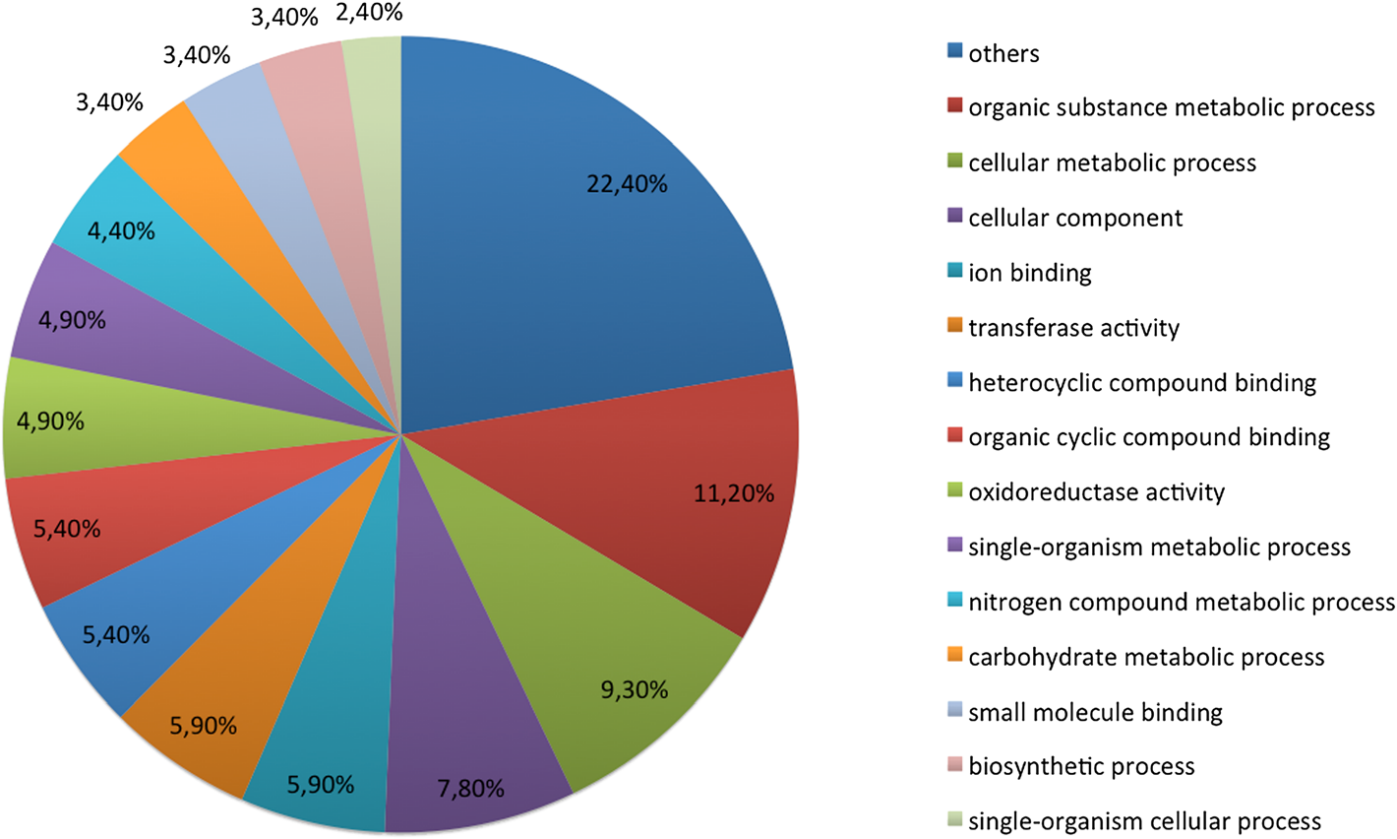
**Pie chart distribution of the Gene Ontology category analysis.**

The PatternLab’s Gene Ontology Explorer module shows the most representative class of proteins according to the Gene Ontology classification. As can be noted in Figure 3, the majority of the proteins were mapped was the “Organic substance metabolic process” (11.2%) GO term which includes proteins belonging to many different biological processes. Other worth-mentionig categories are: transferase activity (5.9%), ion binding (5.9%), and oxireductase activity (4.9%). For the sake of completeness, we used more specific classifications retrieved from Uniprot server to describe each class of proteins in the tables.

### Protein-protein interaction network analysis

We created interaction networks from the proteomic data using the STITCH tool to look for proteins candidates coordinating the response of *C. violaceum* in response to the high concentration of iron. For this, proteins identified exclusively in the iron-responsive proteome and those found to be up-regulated served as input for the interactomes tools.

**Figure 4:**
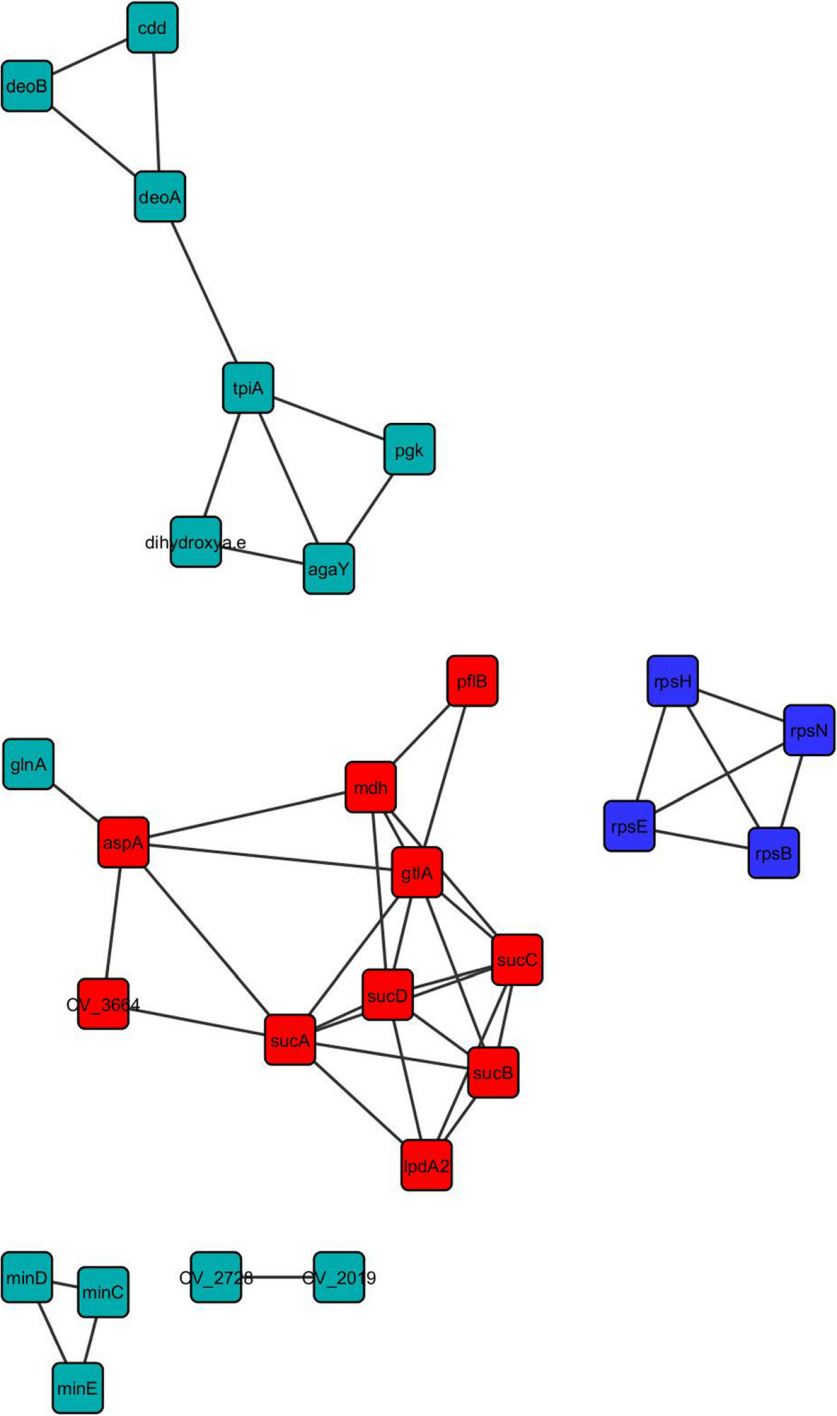
**Protein interaction network of the iron-responsive proteome.** The protein input was the data obtained from the proteomics analysis. Only the proteins that were up-regulated or exclusively identified in the iron responsive proteome were used in the construction of the protein-protein interaction network. The red and blue nodes represents the two clusters obtained with the MCODE plug-in, in which the red ones are the proteins related to energetic metabolism and the blue dots are ribosomal proteins.

Cystoscape’s interactome analysis revealed a network comprised of 27 nodes and 44 edges (Figure 4). The MCODE plug-in identified two main clusters in the network. The first (Red nodes in Figure 4), corresponds to proteins belonging to energetic metabolism; the second is composed of ribosomal proteins (Blue nodes in Figure 4). Indeed, these were two classes of proteins significantly abundant in our data; a possible role of these proteins in response to iron in *C. violaceum* will be discussed further.

Finally, the centrality indexes provided by the CentisScape allowed the identification of the central nodes controlling the communication between the biological processes (Additional file 6). The nodes with high betweenness were the Fumarate Hidratase (AspA) and α-oxoglutarate dehydrogenase (SucA). This indicates the importance of the energetic metabolism in the iron-responsive proteome of *C. violaceum*.

### Proteins with unknown function

Possible roles for the proteins with unknown function from the iron-responsive proteome were investigated using BLAST and CDD (Conserved Domain Database). The identification of conserved domains may be the only clue to estimate the location and function of some proteins, as their domains have a high similarity with previously characterized proteins [28].

Of the five hypothetical ORFs that were up-regulated in the presence of iron, we were unable to identify any conserved domain in two of them (CV_1472 and CV_3099). The ORF CV_4300 contains the USP-like functional domain that is related to stress defense. According to the CDD, the synthesis of proteins in the USP family is increased when the bacteria is exposed to stressful agents, increasing its survival rate during exposure to the agent.

CV_0868 has a domain of unknown function (DUF1842). This protein was previously detected in *C. violaceum* in another proteomic work from our group that studied this bacterium when submitted to hydrogen peroxide (personal communication). Thus, we inferred this protein to be related to a stress response. The HHpred analysis showed that the primary structure of CV_0868 to be associated to the tertiary structure of a Cu-Zn Superoxide Dismutase from different organisms (Additional file 7). Analyzing the genomic context of CV_0868, we observed that this ORF is adjacent to two other ORFs, one encoding a putative SodB (CV_0867) and the other encoding a protein of unknown function (CV_0869). Thus, we hypothesized *C. violaceum* possess a yet non-described operon related to oxidative stress.

To further investigate our assumption, we performed a RT-PCR of the possible new operon. The results showed the presence of an individual transcript comprising the three mentioned ORFs, leading us to suggest that this is, indeed, a new operon (Additional file 8). We further verified this hypothesis by Real Time PCR for the two ORFs comprising the operon (CV_0868 and CV_0869). The expression analysis (Additional file 9) of these two genes showed that after four hours of treatment, an up-regulation of these ORFs could be observed and therefore strongly suggests these proteins to be a response of *C. violaceum* to the iron treatment.

## Discussion

### Energetic metabolism

Energetic Metabolism describes one of the most expressive groups containing up-regulated or exclusively identified proteins in the presence of iron (Table 1 and Additional file 4). Many enzymes from this metabolic pathway have an Fe-S cluster in its active center, which helps to explain the metabolic exchange to pathways in which enzymes do not use iron in their catalytic core. Supporting our results, Nwugo *et al.* [37] observed a boost in the expression of TCA cycle related proteins when exposing *Acinetobacter baumannii* to an iron rich medium.

**Figure 5:**
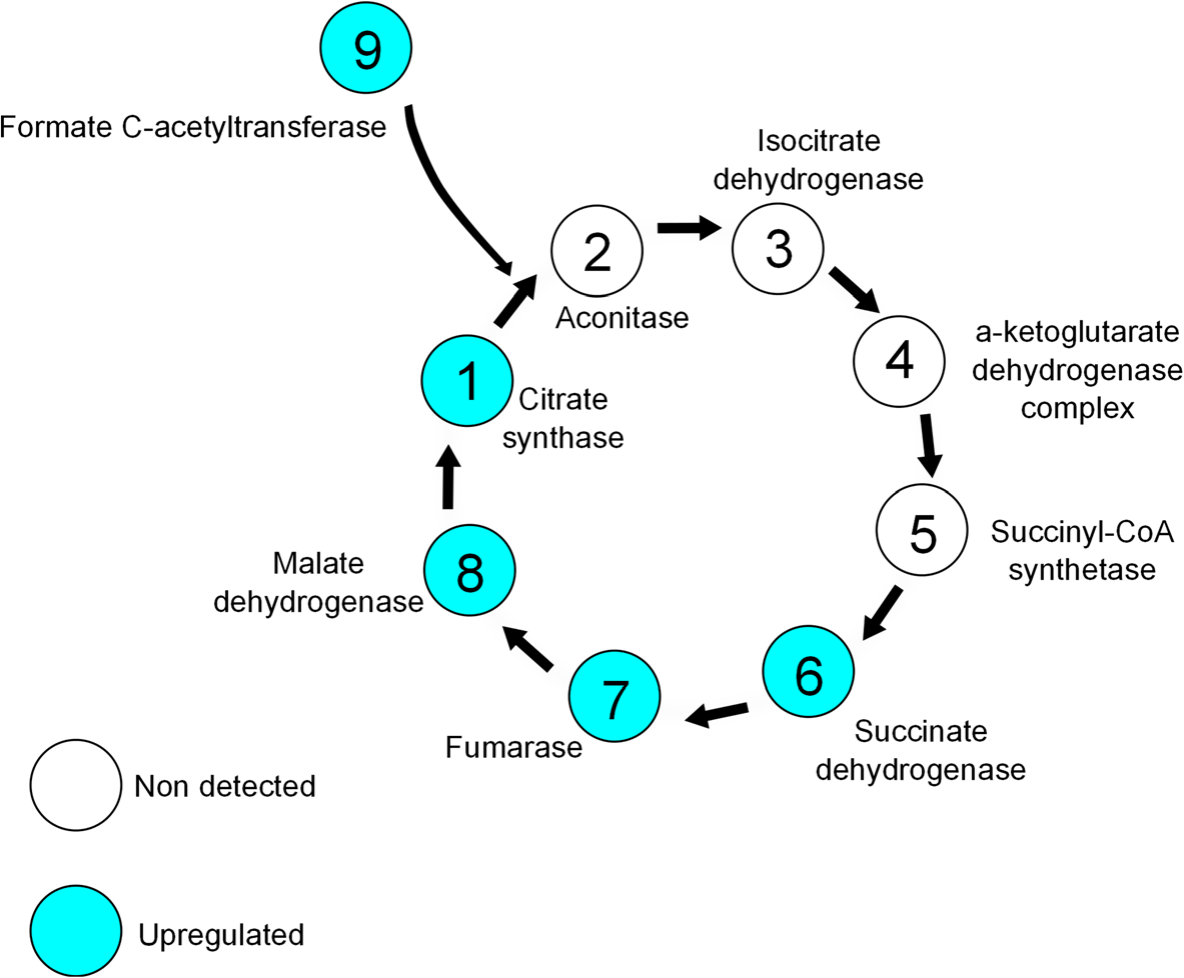
**Integration of energetic metabolism in** ***C. violaceum*** **.** The up-regulation of enzymes with iron in its catalytic center is leading to a boost of the TCA.

Four proteins that were up-regulated after the iron treatment belong to the tricarboxylic acid cycle (SucB, AspA, GtlA, and Mdh). Moreover, the protein Formate c-acetyltransferase (PflB) was up-regulated in the experimental condition. This protein is not known to be directly related to the tricarboxylic acid cycle but could be indirectly playing a role through an anaplerotic pathway. Figure 5 summarizes the energetic metabolism of *C. violaceum* in response to the high concentration of iron used in the culture.

### Stress response

The relationship between iron and oxidative stress is well established. Although iron cannot directly damage biomolecules, it enables the Fenton Reaction, leading to the production of Reactive Oxygen Species (ROS), which harms molecules such as DNA and proteins [38]. The total protein extract from *C. violaceum* was submitted to TAA assay and we observed an increase in the antioxidant activity in the protein extract from the iron treatment (Figure 2C). Additionally, the activity of the antioxidant enzymes Kat and Sod was also increased when the bacterium was cultured in the presence of the metal (Figure 2A and B). Although these assays showed the generation of oxidative stress in the experimental condition, we were unable to detect these enzymes in our mass spectrometry analysis. In fact, we observed the down-regulation of many enzymes clearly related to antioxidant defense (DnaK, HtpG, Dps, and Thioredoxin) (Table 1). Nonetheless, the down-regulation of these enzymes is not necessarily correlated to a decrease in their activities. On the other side, two Glutathione S-transferase proteins were up-regulated in our iron-responsive proteome which suggests that the iron treatment induced an oxidative stress in *C. violaceum*.

Two other proteins that were up-regulated in the iron treatment were a Phasin and an outer membrane protein (OmpW). The former is a granule-associated protein that affects the synthesis and accumulation of Polyhydroxyalkanoates (PHAs) [39]. These polymers contribute to the redox balance and are known to be accumulated when the organism is subject to unfavorable conditions [40]. Thus, to compensate the down-regulation of the usual stress-related proteins, *C. violaceum* might be enhancing the expression of PHAs synthesis-related proteins as Phasin.

OmpW is a porin widely distributed among Gram-negative bacteria and is associated to stress resistance [41]. For example, Gil et al. [42] showed that OmpW becomes up-regulated when *Salmonella enterica* is submitted to Paraquat, a generator of superoxide, promoting the efflux of this ROS. The superoxide anion is yielded as a by-product of electron transporter chain and is a harmful reactive oxygen species. In this way, *C. violaceum* may be up-regulating the synthesis of OmpW to counteract the harmful effects of superoxide, as the main antagonist enzyme of this component (Superoxide dismutase) was not detected in our work.

### General metabolism

General metabolism proteins (replication, translation, cell cycle) were the proteins most abundantly detected in this study, especially between those that were down-regulated in the presence of iron. Indeed, in other proteomics studies, ribosomal proteins, proteins related to the biomolecules synthesis, such as tRNAs, have the greatest representations [43-45]. As one can note from Additional file 2, the iron is inducing a growth arrest in *C. violaceum*. The fact that many proteins from general metabolism were down-regulated in the iron responsive proteome could explain this growth halt.

### Protein-protein interaction network analysis

The bottlenecks from the protein-protein interaction network are key nodes as they represent proteins that connect various functional clusters [35]. Yu and colleagues [35] provide an interesting discussion on importance of the bottleneck proteins in the maintenance of biological systems and that the deletion of some of these proteins may lead to a disruption of signal cascades, and ultimately to cell death. The protein-protein interaction study pinpointed two proteins with high degree of betweenness and node degree inside the iron-responsive network; the AspA and SucA. Both proteins are part of the energetic metabolism, more specifically the Tricarboxylic Acid Cycle, suggesting that the great connectivity between these proteins with the whole iron-responsive network is part of the response of *C. violaceum* to the treatment. The importance of the energetic metabolism to adaptation of many microorganisms in the presence (and absence) of iron has been well established in previous works [14,46].

### An oxidative stress related operon candidate

Our data suggests the identification of a new operon that encompasses the ORFs CV_0867, CV_0868, and CV_0869. The RT-PCR results indicated that all three genes are transcribed as a single transcript. We hypothesize that this operon could be related to oxidative stress mainly because our HHPred analysis indicated CV_0868 as being a Superoxide Dismutase (Sod). Moreover, CV_0868 was up-regulated in the iron-responsive proteome. Further functional characterization experimentation is required to confirm if the protein CV_0868 is a Superoxide Dismutase.

## Conclusions

This is the first proteome study of *Chromobacterium violaceum* in response to a high concentration of iron. The analysis reveals the importance of energetic metabolism and stress proteins to the adaptation of the bacterium to the iron-repleted environment. Most importantly, we identified a new operon, encompassing the ORFs CV_0867, CV_0868 and CV_0869, that is probably related to oxidative stress response. Our data analysis supports the hypothetical protein CV_0868 as a Type C Superoxide Dismutase (SodC). Biochemical studies in our laboratory are being carried out to confirm the dismutation capacity of this protein. The other proteins with unknown function are good candidates to have their role determined in the involvement of iron homeostasis.

### Data availability

All the .raw, .sqt and .sepr files from this work are available for download at http://proteomics.fiocruz.br/dchaves/2014-1.

## Additional files

Additional file 1: **List of all primers used in this work.**

Additional file 2: **Growth curve of** ***C. violaceum*** **cultivated in the absence and in the presence of 9 mM iron.**

Additional file 3: **ACFold analysis comparing the proteins expressed in the iron-responsive proteome and our control condition.** Each protein is represented as a dot in the chart. Red dots are proteins that satisfy neither the variable fold-change cutoff nor the FDR cutoff α =0.05. Green dots are those that satisfy the fold-change cutoff but not α. Orange dots are those that satisfy both the fold-change cutoff and α but are lowly abundant proteins and therefore most likely have their quantitations compromised and can yield artificially low p-values. At the end, blue dots are those that satisfy all statistical filters. Dots in the upper part of the plot correspond to proteins over-expressed in the resection margin while the dots in the bottom are proteins down-regulated after exposition to iron.

Additional file 4: **Proteins exclusively expressed in the iron responsive proteome.** All the proteins were detected in at least two biological replicates.

Additional file 5: **Proteins exclusively expressed in the control condition.** All the proteins were detected in at least two biological replicates.

Additional file 6: **Chart of the centrality analysis showing Betweenness × Node Degree values.** The proteins with higher betweenness and node degree are the ones predicted as the bottlenecks once they interconnect more pathways.

Additional file 7: **Result of the HHpred analysis made with the protein sequence of CV_0868.** As one can see, its probably structure matches with Type C Superoxide Dismutase.

Additional file 8: **Electrophoresis in 1% Agarose of the RT-PCR amplicon.** The result suggests the amplification of a single transcript, proving that the ORFs CV_0869, CV_0868, and CV_0867 encompass an *operon*. C1 – Negative Control (Nuclease Free Water); C2 – Negative Control (RNA that originates the cDNA was used); O – amplicon representing the *operon* (RT-PCR performed with cDNA).

Additional file 9: **qPCR of the genes CV_0868 (A) and the ORF CV_0867 (B), a sod that is in operon with the former.**
